# Using two-step cluster analysis to classify inpatients with primary biliary cholangitis based on autoantibodies: A real-world retrospective study of 537 patients in China

**DOI:** 10.3389/fimmu.2022.1098076

**Published:** 2023-01-04

**Authors:** Dan-Tong Zhao, Hui-Ping Yan, Hui-Yu Liao, Yan-Min Liu, Ying Han, Hai-Ping Zhang, Wei-Ming Zhang, Chun-Yang Huang, Xiu-Hong Liu, Jin-Li Lou, Yan Zhao

**Affiliations:** ^1^ Clinical Laboratory Center and Clinical Research Center for Autoimmune Liver Disease, Beijing You’An Hospital, Capital Medical University, Beijing, China; ^2^ Second Department of Liver Disease Center, Beijing You’An Hospital, Capital Medical University, Beijing, China; ^3^ Department of Clinical Laboratory Diagnosis, Beijing You’An Hospital, Capital Medical University, Beijing, China; ^4^ Clinical Laboratory Center, Beijing Chest Hospital, Capital Medical University, Beijing, China

**Keywords:** two-step cluster analysis, primary biliary cholangitis, autoantibody, real-world study, retrospective study

## Abstract

**Background:**

A variety of autoantibodies have been detected in primary biliary cholangitis (PBC), while the presence of autoantibody clusters and their clinical significance have not been fully understood. We aimed at defining autoantibody clusters and to better understand the clinical features and prognosis of PBC patients based on autoantibody clusters under real-world conditions.

**Methods:**

We retrospectively analyzed 788 inpatients with PBC evaluated between October 2008 and July 2019, and included 537 patients. Nineteen autoantibodies which were measured routinely were investigated for cluster analysis. Two-step clustering, Kaplan-Meier survival, and Cox regression analyses were used.

**Results:**

Five clusters were defined. A cluster of antinuclear antibodies (ANA) and anti-gp210 positive patients were identified with a high rate of cirrhosis at baseline and low survival rate; a cluster of ANA, anti-centromere antibodies (ACA) and/or anti-CENP-B female dominant patients with older disease onset, low level of platelet count at baseline, high rate of hepatic decompensation, and low survival rate was also characterized; and another cluster of anti-mitochondrial antibodies (AMA) and/or AMA-M2, anti-Ro52 and a high rate of anti-gp210 positive patients were identified with a high proportion of male patients and low survival rate. A subgroup of patients with anti-SSA and/or anti-SSB coexists with SjS was also identified; patients with only AMA and/or AMA-M2-positive with a benign clinical outcome and relatively high complication of non-alcoholic fatty liver disease (NAFLD) were also identified. Only anti-gp210 was considered as a significant predictor for poor outcomes especially in patients with cirrhosis.

**Conclusion:**

Clustering methods allow the identification of distinct autoantibody profiles of PBC that form clinical subsets and can be useful for personalized approaches to diagnosis, clinical management, and the prediction of clinical outcomes. Anti-gp210 was the strongest predictive factor for poor outcomes especially in PBC patients with cirrhosis under real-world conditions.

## Introduction

Primary biliary cholangitis (PBC) is a chronic inflammatory autoimmune cholestatic liver disease with a progressive course that may extend over several decades. If left untreated, the disease will develop into end-stage biliary cirrhosis (1, 2). It is most commonly recognized in women in their 5th or 6th decade of life and is characterized by cholestasis in the presence of radiologically normal bile ducts and serological reactivity to anti-mitochondrial antibodies (AMA) or PBC-specific antinuclear antibodies (ANA) (2, 3). Due to its heterogeneous course and outcome, the rate of disease progression varies greatly among individual patients (4).

Serum autoantibodies are crucial tools for the differential diagnosis of PBC and more than 60 autoantibodies have been detected in PBC patients, of which some have been considered to be PBC-specific and have been examined for their diagnostic utility and prognostic values, such as antiglycoprotein (anti-gp) 210, anti-sp100, and anti-centromere antibodies (ACA) (5–7). However, the clustering based on autoantibody profiles and the clinical significance of autoantibody clusters has not been fully understood.

Real-world data are crucial for understanding not only the treatment effectiveness and safety but also disease diagnosis and progression in everyday clinical practice, particularly in patient populations with PBC that may be underrepresented or excluded from clinical trials, such as those with cirrhosis and mixed phenotypes [e.g. overlap autoimmune hepatitis(AIH)] (8, 9). The present study aimed to define the autoantibody clusters and to analyze their correlations with clinical features based on readily available routine measured autoantibodies; moreover, to better understand the prognosis of PBC patients based on autoantibody clusters in a large retrospective cohort under real-world conditions.

## Methods

### Study population

We performed a retrospective cohort analysis of inpatients with discharge diagnosis of PBC who had immune serological investigations of autoantibodies and attended or were followed up at Beijing You’an hospital between October 2008 and July 2019. The follow-up data were collected until September 2019. The diagnosis of PBC was referred to the Chinese clinical practice guidelines (10), the American Association for the Study of Liver Diseases and the European Association for the Study of the Liver practice guidelines for PBC (2, 4). The main criteria consisted of biochemical evidence of cholestasis with an elevation of alkaline phosphatase (ALP) activity; presence of AMA and/or AMA-M2, or other PBC-specific autoantibodies, including sp100 or gp210, if AMA is negative; histopathological evidence of non-suppurative cholangitis, and destruction of small or medium-sized bile ducts (if a biopsy was performed). A liver biopsy was performed only if the patient met liver biopsy recommendations for the diagnosis of PBC, had no liver decompensation and was at low risk of liver biopsy. The histological stages were determined according to Ludwig’s classification (11). Briefly, stage 1 was characterized by inflammatory destruction of intrahepatic small bile ducts, stage 2 by proliferation of bile ductules and/or piecemeal necrosis, stage 3 by fibrosis and/or bridging necrosis, and stage 4 by cirrhosis. Ursodeoxycholic acid (UDCA) therapy was initiated once the diagnosis was made and maintained at a dose of 13-15 mg/kg during follow-up. The patients with concomitant features of AIH, as defined by the current PBC treatment guidelines fulfilled the Paris criteria for PBC-AIH overlap (10, 12). Patients with present chronic hepatitis B, hepatitis A, hepatitis C, and hepatitis E were not included.

### Study design

Baseline demographic and clinical factors (sex, age, disease duration, signs and symptoms, history of comorbidities, physical examination, biochemical, and serological features) were documented on initial presentation. The nature of co-existent diseases affecting the liver and several other autoimmune disorders were recorded. Cirrhosis was assessed by computed tomography, magnetic resonance imaging or ultrasound examinations, and was diagnosed histologically (if available) or clinically in accordance with Chinese guidelines on the management of liver cirrhosis (13). Hepatic decompensation was defined as the occurrence of variceal bleeding, hepatic encephalopathy, or ascites, whichever occurred first. Past hepatitis B virus (HBV) infection was defined as positive antibody to hepatitis B core antigen (anti-HBc) plus negative hepatitis B surface antigen (HBsAg) (14). Inactive HBV carriers were defined by persistent HBsAg, antibody to hepatitis B e antigen (anti-HBe), low-serum HBV DNA, and normal alanine transaminase (ALT) levels (15). The duration of the disease was defined as the time of history from the diagnosis of PBC to the end of the last follow-up. The duration of follow-up was defined as the time from the first visit to the end of the last follow-up prior to analysis of the data, or the date of transplantation or date of death (event) (7, 16). The patients were classified as lost to follow-up if they could not be contacted or no information was available on their medical condition for more than 6 months. The following clinical outcome measures were considered to be of major interest: death (from any cause and liver-related causes), liver transplantation, hepatic decompensation (variceal bleeding, hepatic encephalopathy, or ascites, whichever occurred first), and hepatocellular carcinoma (HCC). The poor outcomes included liver transplantation and/or death, HCC, and hepatic decompensation. Transplant-free survival was defined as survival free of liver-related death, or liver transplantation (17). Adverse outcome-free survival was defined as survival free of poor outcomes.

### Parameters

A total of 19 autoantibodies were investigated for cluster analysis to identify subsets of patients with PBC. All of these antibodies were measured routinely at the Clinical Laboratory Center and Clinical Research Center for Autoimmune Liver Disease of Beijing You’An Hospital using standard procedures. Autoantibodies tests were prescribed on a fixed basis for patients with autoimmune liver disease (AILD), and the kinds of autoantibodies depended on the kit tests panel. ANA, AMA, and ACA which were common found in PBC patients were determined by indirect immunofluorescence (IIF) using Liver Mosaic test kit (EUROIMMUN, Lübeck, Germany), and a titer ≥1:100 was interpreted as positive. AMA-M2 was detected by enzyme-linked immunosorbent assay (ELISA) using AMA-M2 detection kit (Kexin, Shanghai, China), and the results more than 25RU/mL was interpreted as positive. Anti-extractable nuclear antigens (ENAs) including anti-centromere protein B (CENP B), anti-Ro52, anti-SSA, anti-SSB, anti-Sm, anti-nRNP, anti-dsDNA, anti-Rib, anti-His, anti-Nuk, anti-Scl70, and anti-Jo1 were assessed by immunoblot assays using EUROLine ANA Profile test kit (EUROIMMUN, Lübeck, Germany). The AILD-related autoantibodies including anti-gp210, anti-sp100, anti-SLA, anti-LKM1, and anti-LC1 were assessed by line immunoassay (LIA) using Antibody Profile in Autoimmune Liver Diseases test kit (YHLO, Shenzhen, China).

The associations between autoantibody clusters and baseline data of recognized adverse presenting phenotypes were assessed. The parameters included serum hepatic aminotransferases [aspartate aminotransferase (AST) or ALT] above the clinical laboratory upper limit of normal (ULN) [AST ULN=35U/L (women) or 40U/L (men); ALT ULN= 40 U/L (women) or 50U/L (men)], ALP above 1.5×ULN [ALP ULN=135 U/L (women) or 125U/L(men)], serum bilirubin above ULN (=21 µmol/L), serum albumin below the clinical laboratory lower limit of normal (LLN)(=40g/L), serum immunoglobulin (Ig)G/IgA/IgM above ULN (7, 18) (IgG ULN=16.0g/L, IgA ULN=4.0g/L, IgM ULN=2.3g/L), and platelet counts. The associations between individual autoantibodies and clinical features and outcomes of patients with PBC were assessed and the probabilities of transplant-free survivals and adverse outcome-free survivals among autoantibody clusters were compared prior to and following adjustment for age-onset and sex. Furthermore, the prognostic value of autoantibodies associated with clinical outcomes was finally conducted to elucidate the kind of autoantibody, which played a crucial part in the clusters.

### Statistical analysis

All statistical analyses were performed using the statistical package of IBM SPSS Statistics for Windows version 23 (IBM Corporation, Armonk, NY, USA). Due to the large sample size of the present study, a two-step cluster analysis procedure was conducted over the 19 antibodies (16). To assess the quality of the clustering, the silhouette measure of cluster cohesion and separation is used. Silhouette measure utilizes values between –1 ≤ 0 ≤ 1. Higher values indicate a better clustering structure. Since all the antibody variables examined were categorical variables, the log-likelihood distance was selected for the distance measure and Schwarz’s Bayesian Information Criterion (BIC) was used for the clustering criterion in our cohort. Continuous variables were expressed as mean ± s.d., and categorical variables were presented as the number (or percentage) of the subjects.

In order to compare the trends of prevalence of the autoantibodies among different clusters, a Z score transformation of the autoantibody frequencies was performed (19). The Z scores simplify the clinical interpretation due to the mean of 0 and the normal range of -2.0 to +2.0. A Z-score higher than the population mean will exhibit a positive value, whereas a Z-score below the population mean will exhibit a negative value. The higher the deviation of the Z-score from zero (in a positive or negative direction), the greater the magnitude of the deviation from the mean (20). A value that is 2 standard deviations above the mean (the 97.7th percentile) will exhibit a Z-score of +2.0. To determine the presence of significant differences between the clusters, the Kruskal-Wallis H test was used for the assessment of continuous variables with skewness distribution, and the chi-square test was used for the assessment of categorical variables. Binary logistic regression was performed and the odds ratios (ORs) with 95% CIs were used to quantify the relationship between individual autoantibodies and clinical manifestations and outcomes. The probability of survivals of the five clusters of patients was calculated by Kaplan-Meier plots and compared using log-rank tests and Cox regression. Univariate and multivariate analyses of autoantibody clusters and individual autoantibodies were used to determine the association with survival by the stepwise Cox model. The hazard ratio (HR) was calculated by the Cox regression model in both univariate and multivariate analyses. All analyses were two-sided, with *P* values < 0.05 being considered statistically significant. To control for multiple testing, *P* values were corrected (*Pc*) by the number of comparisons, according to the Bonferroni’s inequality method.

## Results

### Description of the cohort

**Figure 1:**
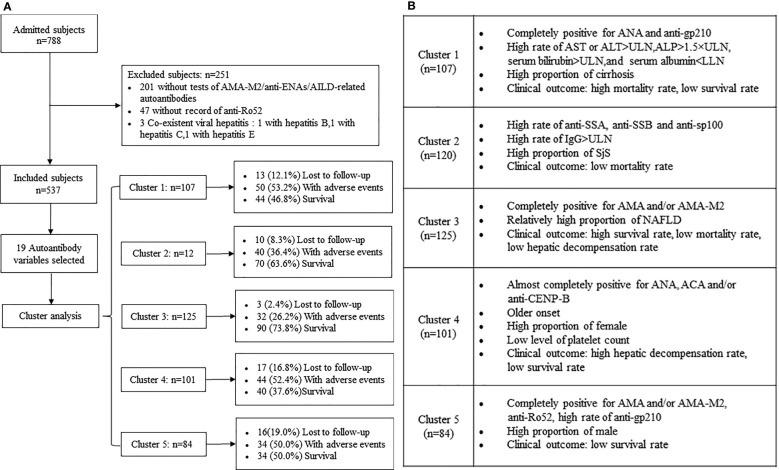
**(A)** Flowchart for selection of patients with PBC and follow-up of the study cohort stratified by autoantibody clusters. **(B)** Main characteristics of the five clusters (clusters 1-5) of patients with PBC.

A total of 788 patients were investigated within the study period and 537 patients with PBC were identified (**Figure 1A**). The percentages of the predominant autoantibody profiles (more than 10%) in patients with PBC were as follows: AMA and/or AMA-M2 (95.0%), ANA (88.1%), anti-Ro52 (39.5%), anti-gp210 (37.4%), ACA and/or anti-CENP-B (24.4%), anti-sp100 (16.6%), and anti-SSA (14.5%). Moreover, anti-LKM1 was not found in the study population (**Table 1**). For the 27 AMA-negative patients, 26 patients were positive for anti-gp210 and/or anti-sp100, only 1 patient was positive for ACA and/or anti-CENP-B with histological diagnosis of PBC (stage 2).

**Table 1:** Comparision and Z Score transformation of autoantibody frequencies in patients with PBC by clusters.

Autoantibody	All patients	Cluster 1		Cluster 2		Cluster 3		Cluster 4		Cluster 5		Overall
	(n=537)	(n=107)	Z score	(n=120)	Z score	(n=125)	Z score	(n=101)	Z score	(n=84)	Z score	*P* value
ANA, n (%)	473 (88.1)	**107 (100.0)**	0.75	113 (94.2)	0.36	80 (64.0)^a^	-1.65	**101 (100.0)**	0.75	72 (85.7)^b^	-0.20	<0.0001
AMA and/or AMA-M2, n (%)	510 (95.0)	90 (84.1)^c^	-1.66	116 (96.7)	0.26	**125 (100.0)**	0.76	95 (94.1)	-0.13	**84 (100.0)**	0.76	<0.0001
ACA and/or anti-CENP-B, n (%)	131 (24.4)	30 (28.0)^a^	0.06	2 (1.7)	-0.56	0 (0.0)	-0.60	**99 (98.0)^a^**	1.72	0 (0.0)	-0.60	<0.0001
Anti-Ro52, n (%)	212 (39.5)	16 (15.0)^a^	-0.73	57 (47.5)	0.11	0 (0.0)^a^	-1.12	55 (54.5)	0.29	**84 (100.0)^a^**	1.46	<0.0001
Anti-SSA, n (%)	78 (14.5)	1 (0.9)	-0.62	**57 (47.5)^a^**	1.65	0 (0.0)	-0.67	19 (18.8)^a^	0.25	1 (1.2)	-0.61	<0.0001
Anti-SSB, n (%)	19 (3.5)	0 (0.0)	-0.52	**17 (14.2)^a^**	1.77	0 (0.0)	-0.52	2 (2.0)	-0.20	0 (0.0)	-0.52	<0.0001
Anti-Sm, n (%)	9 (1.7)	0 (0.0)	-0.53	8 (6.7)	1.77	0 (0.0)	-0.53	1 (1.0)	-0.19	0 (0.0)	-0.53	<0.0001
Anti-nRNP, n (%)	12 (2.2)	0 (0.0)	-0.51	11 (9.2)	1.78	0 (0.0)	-0.51	1 (1.0)	-0.26	0 (0.0)	-0.51	<0.0001
Anti-dsDNA, n (%)	10 (1.9)	0 (0.0)	-0.60	8 (6.7)	1.71	0 (0.0)	-0.60	2 (2.0)	0.09	0 (0.0)	-0.60	<0.0001
Anti-Rib, n (%)	7 (1.3)	0 (0.0)	-0.97	3 (2.5)	0.84	0 (0.0)	-0.97	3 (3.0)	1.20	1 (1.2)	-0.10	0.159
Anti-His, n (%)	4 (0.7)	1 (0.9)	0.20	3 (2.5)	1.67	0 (0.0)	-0.62	0 (0.0)	-0.62	0 (0.0)	-0.62	0.117
Anti-Nuk, n (%)	9 (1.7)	0 (0.0)	-0.58	2 (1.7)	-0.01	0 (0.0)	-0.58	7 (6.9)	1.73	0 (0.0)	-0.58	<0.0001
Anti-Scl70, n (%)	4 (0.7)	1 (0.9)	0.20	3 (2.5)	1.67	0 (0.0)	-0.62	0 (0.0)	-0.62	0 (0.0)	-0.62	0.117
Anti-Jo1, n (%)	2 (0.4)	0 (0.0)	-0.45	0 (0.0)	-0.45	0 (0.0)	-0.45	0 (0.0)	-0.45	2 (2.4)	1.79	0.029
Anti-gp210, n (%)	201 (37.4)	**107 (100.0)^a^**	1.58	41 (34.2)^d^	-0.11	9 (7.2)	-0.81	4 (4.0)	-0.89	40 (47.6)^d^	0.23	<0.0001
Anti-sp100, n (%)	89 (16.6)	5 (4.7)	-0.50	**62 (51.7)^a^**	1.63	0 (0.0)	-0.71	22 (21.8)^a^	0.28	0 (0.0)	-0.71	<0.0001
Anti-SLA, n (%)	6 (1.1)	0 (0.0)	-0.73	1 (0.8)	-0.24	0 (0.0)	-0.73	4 (4.0)	1.70	1 (1.2)	0.00	0.039
Anti-LKM1, n (%)	0 (0.0)	0 (0.0)	/	0 (0.0)	/	0 (0.0)	/	0 (0.0)	/	0 (0.0)	/	/
Anti-LC1, n (%)	4 (0.7)	0 (0.0)	-0.45	0 (0.0)	-0.45	0 (0.0)	-0.45	4 (4.0)	1.79	0 (0.0)	-0.45	0.002

ANA, antinuclear antibodies; AMA, anti-mitochondrial autoantibodies; ACAs, anti-centromere antibodies; Anti-CENP B, anti-centromere protein B; Anti-Ro52, anti-Ro52 antibody; Anti-SSA, anti-SSA antibody; Anti-SSB, anti-SSB antibody; Anti-Sm, anti-Sm antibody; Anti-nRNP, anti-nuclear ribonucleoproteins; Anti-dsDNA, anti-double stranded DNA antibody;Anti-Rib, anti-ribosomal P proteins antibody; Anti-His, anti-histone antibody; Anti-Nuk, anti-Nuk antibody; Anti-Scl70, anti-Scl70 antibody; Anti-Jo1, anti-Jo1 antibody; Anti-gp210, anti-gp210 antibody; Anti-sp100, anti-sp100 antibody; Anti-SLA, anti-soluble liver antigen antibody; Anti-LKM1, anti-liver kidney microsomal type 1 antibody; Anti-LC1, anti-liver cytosol antigen type 1 antibody. ^a^Values significantly different from the other four clusters, all Pc valuses<0.05; ^b^Values significantly different from cluster 1 and cluster 4, all Pc valuses<0.0001; ^c^Values significantly different from cluster 2,cluster 3, and cluster 5; all Pc valuses<0.05; ^d^Values significantly different from cluster 3 and cluster 4, all Pc valuses<0.0001. Bold indicates clusters with the greatest prevalence.

The median age at the time of diagnosis was 55 years (range: 25-87), and 470 (87.5%) patients were women. Serum AST or ALT activities were elevated in 62.1% of the patients (329/530), and 48.0% of the patients (251/523) demonstrated biochemical evidence of cholestasis with serum ALP levels higher than 1.5×ULN. Moreover, 62.4% of the patients (328/526) exhibited serum bilirubin levels higher than ULN. Low serum albumin was found in 73.2% of the patients (385/526). The median platelet counts at baseline were 136×10^9^/L. The percentages of patients who exhibited elevated serum IgM, IgG and IgA levels were 70.2% (327/466), 54.1% (252/466), and 35.0% (163/466) respectively. It is worth noting that 295 (54.9%) of patients presented with cirrhosis at baseline. A total of 173 out of 456 patients (37.9%) exhibited past HBV infection and 2 (0.4%) were inactive HBV carriers. Fatigue, pruritus, and dryness were identified in 44.8% (223/498), 15.5% (77/498), and 8.6% (43/498) of the patients who were enrolled in the present study. The most common comorbidities were hypertension 14.7% (73/498) and diabetes mellitus 12.2% (61/498). The following extrahepatic autoimmune diseases (EHAIDs) were reported: Sjögren’s syndrome (SjS) in 7.2% patients (36/498), rheumatoid arthritis (RA) in 1.8% of patients (9/498), and autoimmune thyroid disease in 0.8% of patients (4/498). A total of 99 (18.4%) patients presented with coexistent diagnoses affecting the liver, 38 (7.1%) of whom had features of clinically classified PBC-AIH overlap; the remaining 61 had coexistent non-autoimmune liver disease [34 presented with drug-induced liver injury, 15 with alcohol-related liver disease, 10 with non-alcoholic fatty liver disease (NAFLD), and 2 with NAFLD and drug-induced liver injury] (**Table 2**).

**Table 2:** Baseline parameters and clinical outcomes of patients with PBC among autoantibody clusters.

	All patients	Cluster 1	Cluster 2	Cluster 3	Cluster 4	Cluster 5	Overall
	(n=537)	(n=107)	(n=120)	(n=125)	(n=101)	(n=84)	*P* value
Baseline parameters							
Age onset (years)	56 ± 11	57 ± 13	54 ± 11	55 ± 12	**59 ± 10^a^**	54 ± 11	0.012
Sex							
Female	470/537 (87.5)	92/107 (86.0)	108/120 (90.0)	106/125 (84.8)	**96/101 (95.0)^b^**	68/84 (81.0)	0.035
Male	67/537 (12.5)	15/107 (14.0)	12/120 (10.0)	19/125 (15.2)	5/101 (5.0)	**16/84 (19.0)^b^**	0.035
AST or ALT>ULN	329/530 (62.1)	**72/104 (69.2)**	72/119 (60.5)	85/125 (68.0)	49/98 (50.0)	51/84 (60.7)	0.034
ALP>1.5×ULN	251/523 (48.0)	**62/103 (60.2)**	50/117 (42.7)	52/123 (42.3)	43/96 (44.8)	44/84 (52.4)	0.039
Serum bilirubin>ULN	328/526 (62.4)	**71/104 (68.3)^c^**	79/117 (67.5)	75/125 (60.0)	46/96 (47.9)	57/84 (67.9)	0.012
Serum albumin<LLN	385/526 (73.2)	**88/104 (84.6)^d^**	82/117 (70.1)	82/125 (65.6)	71/96 (74.0)	62/84 (73.8)	0.024
Platelet count×10^9^/L	154 ± 94	150 ± 92	152 ± 91	184 ± 108	**127 ± 75^e^**	147 ± 86	0.001
IgM>ULN	327/466 (70.2)	71/93 (76.3)	66/101 (65.3)	76/113 (67.3)	54/85 (63.5)	60/74 (81.1)	0.055
IgG>ULN	252/466 (54.1)	50/93 (53.8)	**66/101 (65.3)**	56/113 (49.6)	33/85 (38.8)	47/74 (63.5)	0.002
IgA>ULN	163/466 (35.0)	33/93 (35.5)	45/101 (44.6)	32/113 (28.3)	29/85 (34.1)	24/74 (32.4)	0.163
Cirrhosis	295/537 (54.9)	**74/107 (69.2)^d^**	63/120 (52.5)	48/125 (38.4)	58/101 (57.4)	52/84 (61.9)^f^	<0.0001
Past HBV infection	173/456 (37.9)	41/92 (44.6)	43/103 (41.7)	37/104 (35.6)	29/85 (34.1)	23/72 (31.9)	0.377
Inactive HBV carriers	2/456 (0.4)	0/92 (0.0)	1/103 (1.0)	0/104 (0.0)	1/85 (1.2)	0/72 (0.0)	0.537
Clinical manifestations							
Fatigue	223/498 (44.8)	41/101 (40.6)	56/113 (49.6)	58/117 (49.6)	37/94 (39.4)	31/73 (42.5)	0.390
Pruritus	77/498 (15.5)	20/101 (19.8)	18/113 (15.9)	15/117 (12.8)	11/94 (11.7)	13/73 (17.8)	0.490
Dryness	43/498 (8.6)	10/101 (9.9)	13/113 (11.5)	5/117 (4.3)	11/94 (11.7)	4/73 (5.5)	0.181
Comorbidities							
Hypertension	73/498 (14.7)	16/101 (15.8)	16/113 (14.2)	21/117 (17.9)	11/94 (11.7)	9/73 (12.3)	0.713
Diabetes mellitus	61/498 (12.2)	14/101 (13.9)	12/113 (10.6)	14/117 (12.0)	12/94 (12.8)	9/73 (12.3)	0.968
Sjögren’s syndrome	36/498 (7.2)	5/101 (5.0)	**16/113 (14.2)^g^**	3/117 (2.6)	10/94 (10.6)	2/73 (2.7)	0.002
Rheumatoid arthritis	9/498 (1.8)	1/101 (1.0)	4/113 (3.5)	3/117 (2.6)	0/94 (0.0)	1/73 (1.4)	0.345
Autoimmune thyroid disease	4/498 (0.8)	1/101 (1.0)	1/113 (0.9)	0/117 (0.0)	1/94 (1.1)	1/73 (1.4)	0.848
PBC-AIH overlap	38/537 (7.1)	6/107 (5.6)	10/120 (8.3)	8/125 (6.4)	10/101 (9.9)	4/84 (4.8)	0.620
Drug induced liver injury	36/537 (6.7)	5/107 (4.7)	8/120 (6.7)	14/125 (11.2)	3/101 (3.0)	6/84 (7.1)	0.135
Alcohol-related liver	15/537 (2.8)	4/107 (3.7)	2/120 (1.7)	5/125 (4.0)	1/101 (1.0)	3/84 (3.6)	0.561
Non-alcoholic fatty liver disease	12/537 (2.2)	1/107 (0.9)	3/120 (2.5)	**7/125 (5.6)**	0/101 (0.0)	1/84 (1.2)	0.039
Disease duration	98 ± 54	89 ± 42	99 ± 58	99 ± 55	106 ± 49	96 ± 62	0.121
Duration of follow-up	59 ± 31	52 ± 33	59 ± 29	63 ± 28	60 ± 29	59 ± 33	0.182
Lost to follow-up	59/537 (11.0)	13/107 (12.1)	10/120 (8.3)	3/125 (2.4)^d^	17/101 (16.8)^e^	**16/84 (19.0)^f^**	0.0003
Clinical outcomes							
Survival	278/478 (58.2)	44/94 (46.8)	70/110 (63.6)	**90/122 (73.8)^d,e,f^**	40/84 (47.6)	34/68 (50.0)	<0.0001
Death	130/478 (27.2)	**39/94 (41.5)^d,h^**	22/110 (20.0)	21/122 (17.2)	24/84 (28.6)	24/68 (35.3)	0.0003
Liver transplantation	17/478 (3.6)	5/94 (5.3)	4/110 (3.6)	3/122 (2.5)	3/84 (3.6)	2/68 (2.9)	0.852
Hepatic decompensation	45/478 (9.4)	5/94 (5.3)	12/110 (10.9)	6/122 (4.9)	**16/84 (19.0)^e^**	6/68 (8.8)	0.007
Hepatocellular carcinoma	8/478 (1.7)	1/94 (1.1)	2/110 (1.8)	2/122 (1.6)	1/84 (1.2)	2/68 (2.9)	0.908

AST, aspartate aminotransferase; ALT, alanine transaminase; ULN, upper limit of normal; ALP, alkaline phosphatase; LLN, lower limit of normal; IgM, immunoglobulin M; IgG, immunoglobulin M; IgA, immunoglobulin A; HBV, hepatitis B virus; AIH, autoimmune hepatitis. ^a^Values significantly different between cluster 2 and cluster 4, Pc valuse=0.020; ^b^Values significantly different between cluster 4 and cluster 5, all Pc valuses=0.040; ^c^Values significantly different between cluster 1 and cluster 4, Pc valuse=0.040; ^d^Values significantly different between cluster 1 and cluster 3, all Pc valuses<0.05; ^e^Values significantly different between cluster 3 and cluster 4, all Pc valuses<0.05; ^f^Values significantly different between cluster 3 and cluster 5, all Pc valuses<0.05; ^g^Values significantly different between cluster 2 and cluster 3, Pc valuse=0.010; ^h^Values significantly different between cluster 1 and cluster 2, Pc valuse=0.010. Bold indicates clusters with the greatest prevalence and/or overall P value<0.05.

### Autoantibody clusters and their baseline clinical features

**Figure 2:**
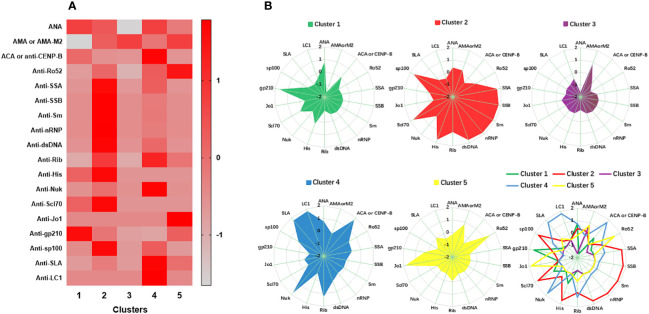
**(A)** Autoantibody clusters of patients with PBC in the study. Heatmap shows the Z Scores of the frequencies for each autoantibody by cluster. The scale on the right denotes Z Scores of antibody frequencies from grey (-2.0, low) to red (+2.0, high). Heatmap rows corresponding to clusters 1, 2, 3, 4 and 5 are indicated. **(B)** Radar plots shows the Z Scores of the frequencies for each autoantibody by cluster. For a given circle each radius represents Z Scores of each autoantibody frequency. Points at the center represent -2.0, whereas points at the perimeter represent +2.0.

The results that emerged from the two-step cluster analysis revealed that the best fit was noted for the five major autoantibody clusters corresponding to the 537 patients. The average silhouette of the model was 0.4. The largest cluster was 125 patients (23.2%), and the smallest cluster was 84 patients (15.6%). The ratio of the cluster sizes (largest cluster to smallest cluster) was 1.49. The autoantibody frequencies and the Z score transformation are shown in **Table 1** and **Figure 2**. Significant differences were noted in the frequencies of 15 autoantibodies, with the exception of anti-Rib, anti-His, and anti-Scl70. Only autoantibodies that were present in at least 50% of patients in a cluster were considered to be a strong characteristic of the cluster (21). Low prevalence (less than 10%) autoantibodies were not considered an important characteristic of this or any other cluster.

Cluster 1 (n=107, 19.9%). The patients were all positive for both ANA and anti-gp210 and exhibited the highest prevalence with regard to biochemical markers of the adverse presenting phenotypes-AST or ALT >ULN (69.2%, *P*=0.034) and ALP >1.5×ULN (60.2%, *P*=0.039) among the five clusters. The percentage of serum bilirubin >ULN [68.3% vs. 47.9% (cluster 4, *Pc*=0.04)], serum albumin <LLN [84.6% vs. 65.6% (cluster 3, *Pc*=0.01)] and cirrhosis [69.2% vs. 38.4% (cluster 3, *Pc*<0.0001)] were higher compared with those noted in the other clusters (**Tables 1**, **2**; **Figure 1B**).

Cluster 2 (n=120, 22.3%). The patients were characterized by the highest prevalence of anti-SSA (47.5%), anti-SSB (14.2%), and anti-sp100 (51.7%) compared with those of the other clusters (the overall *P* values and *Pc* values were all <0.05). The main distinctive baseline clinical feature of this cluster was the lowest median age compared with other clusters (*P*=0.012), notably compared with cluster 4 (*Pc*=0.02). The percentage of IgG >ULN (65.3%) was the highest among the five clusters examined (*P*=0.002). The most common associated condition of cluster 2 was the high prevalence of SjS compared with other clusters (14.2%, *P*=0.002) (**Tables 1**, **2**; **Figure 1B**).

Cluster 3 (n=125, 23.3%). The patients were all positive for AMA and/or AMA-M2 with the lowest frequency of ANA (64.0%) compared with other clusters (overall *P* values and *Pc* values were all <0.0001). In addition to the dominant antibodies, the prevalence of anti-gp210 (7.2%) in cluster 3 was less than 10% and other antibodies were negative. The percentage of serum albumin <LLN (65.6%) was the lowest among the five clusters (*P*=0.024). The baseline median levels of the platelet count in cluster 3 were higher than those noted in the other clusters (*P*=0.001) and was significantly higher than that of cluster 4 (*Pc*<0.0001). Moreover, the percentage of cirrhosis of cluster 3 (38.4%) was the lowest among the five clusters (*P*<0.0001) and the prevalence of SjS in this cluster was significantly lower than that noted in the other clusters (*P*=0.002). However, the prevalence of NAFLD (5.6%) in cluster 3 was the greatest among the five clusters (*P*=0.039) (**Tables 1**, **2**; **Figure 1B**).

Cluster 4 (n=101, 18.8%). All patients in this cluster were positive for ANA and were characterized by the highest prevalence of ACA and/or anti-CENP-B (98.0%) compared with that of other clusters (overall *P* values and *Pc* values were all <0.0001). The main distinctive baseline clinical feature of this cluster was the highest median age compared with that of other clusters (*P*=0.012). The proportion of females was higher than that noted in other clusters (*P*=0.035). The patients in this cluster exhibited significantly lowest prevalence of serum bilirubin >ULN compared with that of other clusters (*P*=0.012). However, cluster 4 exhibited the lowest baseline median levels of the platelet count among the five clusters (*P*=0.001) (**Tables 1**, **2**; **Figure 1B**).

Cluster 5 (n=84, 15.6%). The patients were all positive for both AMA and/or M2 and anti-Ro52. The frequency of anti-gp210 was lower than that noted in cluster 1 (47.6% vs. 100.0%, *Pc*<0.0001) and significantly higher than that noted in cluster 3 (47.6% vs. 7.2%, *Pc*<0.0001) and cluster 4 (47.6% vs. 4.0%, *Pc*<0.0001). The proportion of male patients was the highest in cluster 5 compared with that of other clusters (*P*=0.035), notably cluster 4 (19.0% vs. 5.0%, *Pc*=0.040). The percentage of cirrhosis of this cluster was higher than that of cluster 3 (61.9% vs. 38.4%, *Pc*=0.001). (**Tables 1**, **2**; **Figure 1B**).

### Associations between individual autoantibodies and clinical features and outcomes

Predominant autoantibodies or over-represented clinical features and outcomes of each clusters as identified by cluster analysis were listed together for easier visualization (**Tables 1**, **2**; **Figure 1B**). The findings were highlighted in bold font in tables which indicated they were with the greatest prevalence among clusters and/or overall *P* value <0.05. This analysis was performed without prior clustering. The separate association analysis between individual autoantibodies and clinical features was partly consistent with the previous observations derived from the cluster analysis (**Table 3**).

**Table 3:** Associations between individual autoantibodies, clinical features and outcomes of patients with PBC .

	ANA			AMA and/or AMA-M2			ACA and/or anti-CENP-B			Anti-Ro52			Anti-SSA			Anti-SSB			Anti-gp210			Anti-sp100		
Clinincal manifestation	OR	95CI	*P*	OR	95CI	*P*	OR	95CI	*P*	OR	95CI	*P*	OR	95CI	*P*	OR	95CI	*P*	OR	95CI	*P*	OR	95CI	*P*
Age onset	**0.997**	**0.964-1.032**	**0.882**	0.963	0.916-1.013	0.144	**1.011**	**0.983-1.040**	**0.455**	0.999	0.977-1.022	0.931	0.998	0.966-1.032	0.917	0.978	0.904-1.058	0.576	0.985	0.963-1.008	0.200	1.029	0.999-1.060	0.056
Female	**0.525**	**0.220-1.254**	**0.147**	**2.733**	**0.310-24.112**	**0.365**	**0.588**	**0.228-1.519**	**0.273**	**0.934**	**0.453-1.926**	**0.854**	0.165	0.021-1.311	0.088	0	0	0.998	1.376	0.682-2.775	0.372	0.948	0.380-2.364	0.909
AST or ALT>ULN	**0.901**	**0.421-1.928**	**0.788**	2.511	0.808-7.805	0.112	0.824	0.448-1.515	0.532	0.701	0.423-1.162	0.169	0.436	0.207-0.916	0.028	1.745	0.239-12.752	0.583	**1.046**	**0.622-1.761**	**0.865**	1.009	0.520-1.958	0.980
ALP>1.5×ULN	**1.647**	**0.836-3.244**	**0.149**	0.754	0.248-2.294	0.619	1.032	0.580-1.835	0.916	1.555	0.970-2.493	0.067	0.847	0.423-1.696	0.639	0.957	0.213-4.294	0.945	**1.750**	**1.096-2.796**	**0.019**	0.739	0.402-1.361	0.332
Serum bilirubin>ULN	**0.660**	**0.312-1.397**	**0.277**	0.377	0.108-1.315	0.126	0.463	0.250-0.856	0.014	0.837	0.502-1.395	0.494	1.705	0.800-3.633	0.167	5.405×10^7^	0	0.995	**1.150**	**0.689-1.918**	**0.594**	1.949	0.973-3.902	0.060
Serum albumin<LLN	**0.579**	**0.241-1.392**	**0.222**	0.203	0.023-1.816	0.154	1.684	0.808-3.509	0.164	0.905	0.493-1.658	0.746	1.401	0.562-3.496	0.469	3.335×10^7^	0	0.995	**1.225**	**0.665-2.255**	**0.515**	0.741	0.338-1.623	0.454
Platelet count×10^9^/L	**1.000**	**0.996-1.004**	**0.855**	0.997	0.991-1.003	0.384	**0.995**	**0.991-0.999**	**0.021**	0.997	0.994-1.000	0.036	1.000	0.996-1.005	0.870	1.001	0.992-1.009	0.894	1.000	0.998-1.003	0.729	1.002	0.998-1.005	0.296
IgG>ULN	2.074	0.826-5.211	0.121	3.813	0.899-16.181	0.070	0.086	0.033-0.221	<0.0001	**1.177**	**0.684-2.027**	**0.556**	4.542	2.162-9.545	<0.0001	2.436	0.647-9.173	0.188	0.864	0.497-1.501	0.603	1.164	0.588-2.304	0.662
Cirrhosis	0.806	0.333-1.949	0.632	0.752	0.151-3.759	0.729	0.556	0.242-1.277	0.166	1.128	0.593-2.148	0.713	**0.906**	**0.355-2.316**	**0.837**	**1.447**	**0.167-12.556**	**0.737**	1.749	0.921-3.322	0.088	**1.066**	**0.466-2.439**	**0.879**
SjS	1.331	0.294-6.034	0.711	2.156	0.249-18.663	0.485	1.315	0.485-3.566	0.590	1.673	0.728-3.846	0.226	**6.501**	**2.600-16.253**	**<0.0001**	**27.958**	**4.954-157.777**	**0.0002**	0.877	0.364-2.113	0.770	**1.265**	**0.441-3.631**	**0.662**
NAFLD	0.233	0.059-0.913	0.037	0.200	0.018-2.257	0.193	0	0	0.999	0.284	0.035-2.311	0.239	3.248	0.606-17.424	0.169	0	0	0.999	0.879	0.216-3.574	0.857	**0.546**	**0.065-4.555**	**0.576**
Clinical outcomes																								
Survival	1.167	0.312-4.366	0.819	**0**	**0**	**0.998**	0.781	0.201-3.034	0.721	0.557	0.194-1.605	0.279	1.286	0.241-6.855	0.768	0.428	0.032-5.633	0.519	0.61	0.214-1.737	0.354	0.684	0.189-2.472	0.562
Dead	**2.589**	**0.641-10.458**	**0.182**	0	0	0.998	1.579	0.421-5.913	0.498	0.635	0.222-1.812	0.396	0.735	0.137-3.938	0.719	0.363	0.028-4.636	0.436	**1.039**	**0.368-2.935**	**0.943**	0.571	0.158-2.066	0.393
Hepatic decompensation	**1.343**	**0.272-6.642**	**0.718**	0	0	0.998	**1.685**	**0.391-7.262**	**0.484**	0.680	0.205-2.260	0.530	1.607	0.267-9.655	0.604	0.201	0.007-5.889	0.352	0.644	0.193-2.152	0.474	1.167	0.275-4.948	0.834

AST, aspartate aminotransferase; ALT, alanine transaminase; ULN, upper limit of normal; ALP, alkaline phosphatase; LLN, lower limit of normal; IgG, immunoglobulin M;SjS, Sjogren’s syndrome; NAFLD, nonalcoholic fatty liver disease; OR, odds ratios; CI, confidence interval; ANA, antinuclear antibodies; AMA, anti-mitochondrial autoantibodies; ACAs, anti-centromere antibodies; Anti-CENP B, anti-centromere protein B; Anti-Ro52, anti-Ro52 antibody; Anti-SSA, anti-SSA antibody; Anti-SSB, anti-SSB antibody; Anti-gp210, anti-gp210 antibody; Anti-sp100, anti-sp100 antibody. Bold text denotes associations between the over-represented clinical manifestations of the same cluster from cluster analysis (**Tables 1** and **2)**

ANA was the predominant autoantibody of each cluster, and was marginally associated with patients combined with NAFLD (OR: 0.233, 95% CI: 0.059-0.913, *P*=0.037). AMA and/or AMA-M2 were not associated with all over-represented clinical manifestations and outcomes of each cluster as identified by cluster analysis. This was consistent with the observations from previous cluster analysis since AMA and/or AMA-M2 were the predominant autoantibodies of all clusters, even in cluster 1 with the lowest prevalence of 84.1% compared with cluster 2 (96.7%, *Pc*=0.01), cluster 3 (100.0%, *Pc*<0.0001), and cluster 5 (100.0%, *Pc*<0.0001).

ACA and/or anti-CENP-B were the predominant autoantibodies of cluster 4 and were associated with lower serum bilirubin (OR: 0.463, 95% CI: 0.250-0.856, *P*=0.014), lower platelet count (OR: 0.995,95% CI: 0.991-0.999, *P*=0.021), lower levels of IgG (OR: 0.086, 95% CI: 0.033-0.221, *P*<0.0001). Notably, lower platelet count was the representative clinical manifestation of the same cluster during cluster analysis. Anti-Ro52 was associated with lower platelet count (OR: 0.997, 95% CI: 0.994-1.000, *P*=0.036), and lower platelet count was the over-representative clinical manifestations of cluster 4.

Anti-SSA and anti-SSB were the predominant autoantibodies of cluster 2 and were associated with SjS [anti-SSA (OR: 6.501, 95% CI: 2.600-16.253, *P*<0.0001), anti-SSB (OR: 27.958, 95% CI: 4.954-157.777, *P*=0.0002)]. These were the representative clinical manifestations of the same cluster during cluster analysis. Furthermore, anti-SSA was associated with lower levels of AST or ALT (OR: 0.436, 95% CI: 0.207-0.916, *P*=0.028), and higher levels of IgG (OR: 4.542, 95% CI: 2.162-9.545, *P*<0.0001). Similarly, anti-gp210 which was the characteristic autoantibody of cluster 1 was associated with higher levels of ALP (OR: 1.750, 95% CI: 1.096-2.796, *P*=0.019), one of the representative clinical manifestations of that cluster. Although anti-sp100 was one of the over-representative autoantibodies of cluster 2, it was not significantly associated with the presence of predominant clinical features of the same cluster.

### Prognostic significance of the clusters

In this study cohort, the median duration of the disease was 92 months, and the median duration of the follow-up period was 60 months. During the follow-up period, 59 patients (11.0%) were lost to follow-up, 41.8% of the patients (200/478) developed adverse outcomes, including 130 patients (27.2%) died due to liver-related causes or other causes. A total of 17 patients (3.6%) underwent liver transplantation at the end of the follow-up period or prior to death, whereas 45 patients (9.4%) presented with hepatic decompensation, and 8 patients (1.7%) with hepatocellular carcinoma. The remaining 278 patients (58.2%) were still alive at the end of the follow-up period. Cluster 3 exhibited the highest survival rate (73.8%, *P*<0.0001), which was notably higher than that of cluster 1 (46.8%, *Pc*=0.0005), cluster 4 (47.6%, *Pc*=0.001), and cluster 5 (50.0%, *Pc*=0.010). Similarly, the mortality rate in cluster 1 was the highest compared with that of other clusters (41.5%, *P*=0.0003), notably compared with that of cluster 2 (41.5% vs. 20.0%, *Pc*=0.010) and cluster 3 (41.5% vs. 17.2%, *Pc*=0.0008). The rate of hepatic decompensation was the highest in cluster 4 (19.0%, *P*=0.007) among the five clusters, notably compared with that of cluster 3 (4.9%, *Pc*=0.010). Finally, no differences were noted in the liver transplantation rate as well as the incidence of hepatocellular carcinoma among the five clusters (**Table 2**).

**Figure 3:**
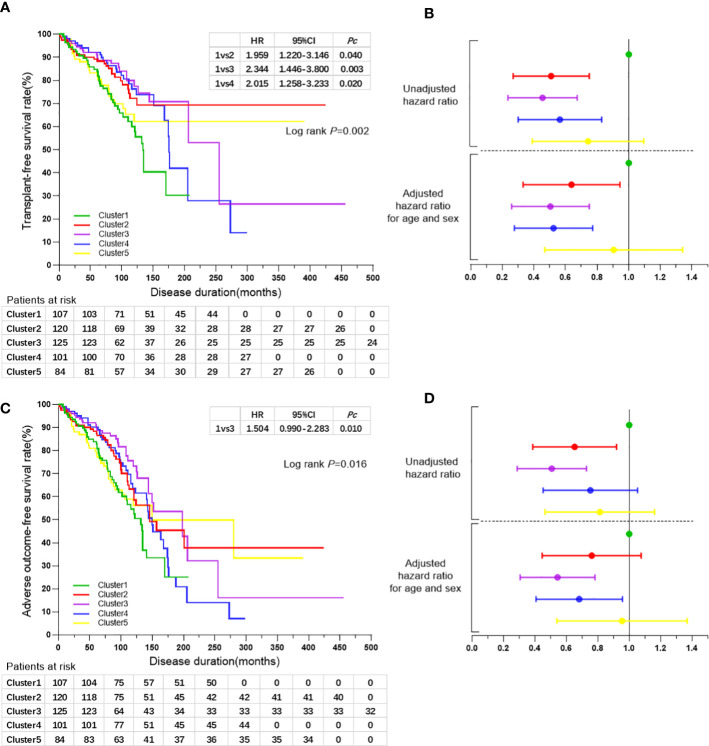
**(A)** Kaplan-Meier survival curves for the comparisons of transplant-free survival rates among the five clusters of patients with PBC. **(B)** Forest plot showing hazard ratios of liver-related death or liver transplantation rates and 95% confidence intervals for the five clusters of patients with PBC. Black line perpendicular to the horizontal axis shows the hazard ratio for the reference group of cluster 1. The horizontal broken line indicates the diving line for hazard ratios of liver-related death or liver transplantation rates before and after adjusted for age and sex. Colors represent the different clusters as indicated in **(A, C)** Kaplan-Meier survival curves for the comparisons of adverse outcome-free survival rates among the five clusters of patients with PBC. **(D)** Forest plot showing hazard ratios of adverse outcome rates and 95% confidence intervals for the five clusters of patients with PBC. Black line perpendicular to the horizontal axis shows the hazard ratio for the reference group of cluster 1. The horizontal broken line indicates the diving line for hazard ratios of adverse outcome rates before and after adjusted for age and sex. Colors represent the different clusters as indicated in **(A)**.

Kaplan-Meier analyses indicated that patients in cluster 1 exhibited a higher risk of liver-related death or liver transplantation compared with those in the other four clusters (all log-rank *P*=0.002). The log-rank test indicated an increased risk of liver-related death or liver transplantation in cluster 1 compared with that of cluster 2 (HR 1.959, 95% CI 1.220-3.146, *Pc*=0.040), cluster 3 (HR 2.344, 95% CI 1.446-3.800, *Pc*=0.003), and cluster 4 (HR 2.015, 95% CI 1.258-3.233, *Pc*=0.020), respectively (**Figure 3A**). Cox regression analyses following adjustment for age-onset and sex indicated a reduced risk of liver-related death or liver transplantation in cluster 2 (HR 0.589, 95% CI 0.359-0.966, *P*=0.036), cluster 3 (HR 0.465, 95% CI 0.281-0.768, *P*=0.003) and cluster 4 (HR 0.485, 95% CI 0.298-0.789, *P*=0.004) compared with that of cluster 1 (**Table 4**; **Figures 3A, B**).

**Table 4:** Cox regression analyses of liver-related death or liver transplantation, and adverse outcome between autoantibody clusters of patients with PBC.

	Univariate analysis			Multivariate analysis					
				Adjusted for age onset			Adjusted for age onset and sex		
	HR	95%CI	*P*	HR	95%CI	*P*	HR	95%CI	*P*
Liver-related death or liver transplantation									
Cluster1	Reference			Reference			Reference		
Cluster2	0.471	0.290-0.767	0.002	0.581	0.358-0.962	0.035	0.589	0.359-0.966	0.036
Cluster3	0.419	0.255-0.690	0.001	0.466	0.282-0.770	0.003	0.465	0.281-0.768	0.003
Cluster4	0.523	0.323-0.847	0.008	0.481	0.297-0.779	0.003	0.485	0.298-0.789	0.004
Cluster5	0.687	0.421-1.120	0.132	0.839	0.512-1.377	0.488	0.835	0.508-1.372	0.477
Adverse outcome									
Cluster1	Reference			Reference			Reference		
Cluster2	0.617	0.406-0.936	0.023	0.721	0.472-1.101	0.130	0.718	0.470-1.096	0.125
Cluster3	0.475	0.305-0.742	0.001	0.505	0.323-0.789	0.003	0.509	0.325-0.797	0.003
Cluster4	0.713	0.474-1.071	0.103	0.654	0.435-0.984	0.042	0.645	0.427-0.973	0.037
Cluster5	0.763	0.492-1.184	0.227	0.885	0.568-1.380	0.590	0.894	0.573-1.396	0.624

HR, hazard ratio; CI, confidence interval.

The risk of adverse outcome was increased for patients in cluster 1 compared with that in the other four clusters (all log-rank *P*=0.016), notably in cluster 3 as determined by the log-rank test (HR 1.504, 95% CI 0.990-2.283, *P*=0.010). Cox regression analysis following adjustment for age-onset and sex indicated a reduced risk of adverse outcomes in cluster 3 (HR 0.509, 95% CI 0.325-0.797, *P*=0.003) and cluster 4 (HR 0.645, 95% CI 0.427-0.973, *P*=0.037) compared with that of cluster 1 (**Table 4**; **Figures 3C, D**).

### Univariate and multivariate risk factor analysis of autoantibodies in patients with PBC

**Figure 4:**
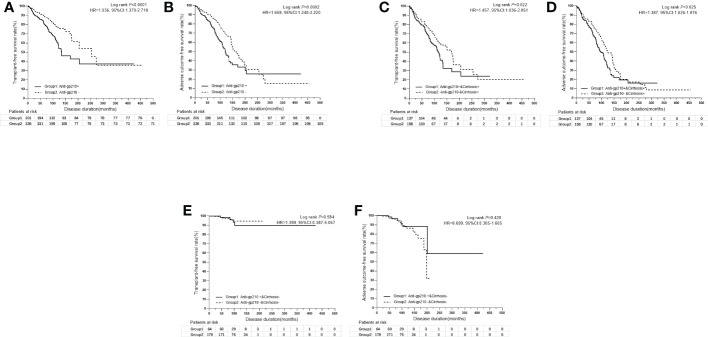
**(A)** Kaplan-Meier survival curves for the comparison of transplant-free survival rates between anti-gp210 positive PBC patients and anti-gp210 negative PBC patients. **(B)** Kaplan-Meier survival curves for the comparison of adverse outcome-free survival rates between anti-gp210 positive PBC patients and anti-gp210 negative PBC patients. **(C)** Kaplan-Meier survival curves for the comparison of transplant-free survival rates between anti-gp210 positive PBC patients with cirrhosis and anti-gp210 negative PBC patients with cirrhosis at baseline. **(D)** Kaplan-Meier survival curves for the comparison of adverse outcome-free survival rates between anti-gp210 positive PBC patients with cirrhosis and anti-gp210 negative PBC patients with cirrhosis at baseline. **(E)** Kaplan-Meier survival curves for the comparison of transplant-free survival rates between anti-gp210 positive PBC patients without cirrhosis and anti-gp210 negative PBC patients without cirrhosis at baseline. **(F)** Kaplan-Meier survival curves for the comparison of adverse outcome-free survival rates between anti-gp210 positive PBC patients without cirrhosis and anti-gp210 negative PBC patients without cirrhosis at baseline.

By using univariate analysis, anti-SSB (*P*=0.016), anti-Rib (*P*=0.002), anti-His (*P*=0.030), anti-Jo1 (*P*=0.043), anti-gp210 (*P*=0.001), and anti-sp100 (*P*=0.021) were shown to be significant risk factors for liver transplantation and/or liver-related death; however, anti-gp210 (*P*=0.0004) was the only significant risk factor for the incidence of adverse outcomes. Multivariate analysis was performed by including all the autoantibodies, which were assessed by the stepwise Cox model. The results indicated that only anti-gp210 was considered to be a significant predictor for both liver transplantation and/or liver-related death (HR 1.964, 95% CI 1.421-2.715, *P*<0.0001), and for adverse outcomes (HR 1.680, 95% CI 1.272-2.218, *P*=0.0003) (**Table 5**). PBC patients with or without anti-gp210 exhibited significant differences in the long-term prognosis. The cumulative 5-year transplant-free survival rates of patients with anti-gp210 were 76.6% compared with 84.8% for anti-gp210 negative cases (*P*<0.0001) (**Figure 4A**). Similarly, the cumulative 5-year adverse outcome-free survivals rates of patients with anti-gp210 were 76.6% compared with 84.8% for anti-gp210 negative cases (*P*=0.0002) (**Figure 4B**). The differences of transplant-free survival rates, as well as adverse outcome-free survival rates between anti-gp210 positive PBC patients with cirrhosis and anti-gp210 negative PBC patients with cirrhosis at baseline were statistically significant (*P* values were all <0.05) (**Figures 4C, D**). However, for PBC patients without cirrhosis, there was no significant difference in transplant-free survival rates and adverse outcome-free survival rates between anti-gp210 positive patients and anti-gp210 negative patients (*P* values were all >0.05) (**Figures 4E, F**).

**Table 5:** Risk of of autoantibodies associated with liver-related death or liver transplantation, and adverse outcome in patients with PBC.

	Univariate analysis						Multivariate analysis					
	Liver-related death or liver transplantation			Adverse outcome			Liver-related death or liver transplantation			Adverse outcome		
Autoantibody	HR	95%CI	*P*	HR	95%CI	*P*	HR	95%CI	*P*	HR	95%CI	*P*
ANA	1.139	0.641-2.026	0.657	1.131	0.694-1.843	0.620						
AMA and/or AMA-M2	0.651	0.334-1.271	0.208	0.688	0.388-1.221	0.201						
ACA and/or anti-CENP-B	1.171	0.793-1.730	0.427	1.208	0.867-1.685	0.264						
Anti-Ro52	0.951	0.669-1.352	0.778	1.050	0.778-1.419	0.749						
Anti-SSA	1.071	0.599-1.914	0.818	1.326	0.841-2.090	0.224						
Anti-SSB	2.793	1.213-6.433	0.016	1.675	0.7568-3.653	0.195						
Anti-Sm	0.468	0.072-3.064	0.428	0.296	0.053-1.645	0.164						
Anti-nRNP	0.517	0.059-4.548	0.552	0.928	0.198-4.341	0.924						
Anti-dsDNA	0.980	0.304-3.158	0.973	0.680	0.212-2.179	0.516						
Anti-Rib	4.806	1.776-13.006	0.002	2.448	0.902-6.644	0.079						
Anti-His	13.818	1.285-148.620	0.030	4.316	0.644-28.914	0.132						
Anti-Nuk	<0.0001	0-6.891 × 10^139^	0.937	0.213	0.039-1.172	0.075						
Anti-Scl70	4.030	0.972-16.711	0.055	1.549	0.301-7.977	0.601						
Anti-Jo1	8.108	1.071-61.395	0.043	6.387	0.858-47.566	0.070						
Anti-gp210	1.853	1.306-2.627	0.001	1.716	1.271-2.315	0.0004	1.964	1.421-2.715	<0.0001	1.680	1.272-2.218	0.0003
Anti-sp100	0.530	0.309-0.908	0.021	0.715	0.469-1.089	0.118						
Anti-SLA	0.590	0.081-4.297	0.602	0.780	0.190-3.199	0.730						
Anti-LC1	3.319	0.423-26.050	0.254	2.973	0.682-12.957	0.147						

ANA, antinuclear antibodies; AMA, anti-mitochondrial autoantibodies; ACAs, anti-centromere antibodies; Anti-CENP B, anti-centromere protein B; Anti-Ro52, anti-Ro52 antibody; Anti-SSA, anti-SSA antibody; Anti-SSB, anti-SSB antibody; Anti-Sm, anti-Sm antibody; Anti-nRNP, anti-nuclear ribonucleoproteins; Anti-dsDNA, anti-double stranded DNA antibody;Anti-Rib, anti-ribosomal P proteins antibody; Anti-His, anti-histone antibody; Anti-Nuk, anti-Nuk antibody; Anti-Scl70, anti-Scl70 antibody; Anti-Jo1, anti-Jo1 antibody; Anti-gp210, ant-gp210 antibody; Anti-sp100, anti-sp100 antibody; Anti-SLA, anti-soluble liver antigen antibody; Anti-LC1, anti-liver cytosol antigen type 1 antibody; HR, hazard ratio; CI, confidence interval.

## Discussion

PBC is a chronic, cholestatic, autoimmune disease with a variable presentation. Patient presentation and clinical course can be diverse, and one or more autoantibodies may be detected in each patient. However, the presence of specific clusters of autoantibodies and their associations with clinical features have not been investigated. Furthermore, an increasing demand exists for a future classification that combines these different patterns, in order to personalize approaches to diagnosis and clinical management (17, 22). This is the first study, which used an unsupervised clustering technique to evaluate the autoantibody profiles associated with PBC in a large cohort of Chinese patients from a single center under real-world conditions. All autoantibodies investigated in this study were readily available and were measured routinely in the majority of medical centers found in China.

In addition to AMA and PBC-specific ANA, including anti-gp210 and anti-sp100, other ANAs, such as anti-Ro52, ACA and/or anti-CENP-B, and anti-SSA were also commonly present in PBC patients. With the exception of very few autoantibodies, the pathogenic roles of autoantibodies in PBC are not clear (6, 23, 24). In this exploratory cluster analysis, the present study revealed five different clusters according to the 19 autoantibodies. The five clusters were characterized by peculiar clinical features which could be distinguished from each other. Anti-gp210 and a high proportion of cirrhosis were the over-represented clinical features in cluster 1, which could explain the poor prognosis of cluster 1. Indeed, liver cirrhosis is a well-known predictor of worse prognosis in PBC (25). To investigate the prognostic value of anti-gp210, we performed Kaplan-Meier survival analysis by risk stratification based on cirrhosis at baseline. We confirmed and extended the observation by demonstrating that anti-gp210 antibody was the strongest predictive factor among autoantibodies for both liver-related death or liver transplantation as well as adverse outcome by multivariate analysis (6, 7). We confirmed the finding that anti-gp210 positive PBC patients had significantly worse outcomes than anti-gp210 negative PBC patients only in the group of patients with cirrhosis at baseline (18). Anti-gp210 indeed could predict a worse prognosis, especially when combined with portal hypertension-related risk factors, such as the presence of cirrhosis at baseline (18). However, for PBC patients without cirrhosis at baseline, the prognostic value of anti-gp210 was not outstanding.

Although ACA and/or anti-CENP-B were not found to be significant predictive factors in the progression to develop a complication of portal hypertension in this analysis, they were associated with lower levels of baseline serum bilirubin, platelet count, and IgG, which was in accordance with the main characteristics of cluster 4. Cluster 4 with the over representative antibody of ACA and/or anti-CENP-B was characterized by a high rate of hepatic decompensation during the follow-up period, which verified the prognostic value of ACA from another perspective (6). Anti-Ro/SSA antibodies are among the most commonly detected autoantibodies in routine screening for autoimmune diseases (26). Granito et al. (27) demonstrated that anti-SS-A/Ro-52kD positive PBC patients exhibited a more advanced histological stage and higher serum levels of bilirubin and IgM at the time of diagnosis compared with negative patients and suggested that further studies with adequate follow-up time periods should be performed. Herein, anti-Ro52 with the overall prevalence of 39.5% was not the predictor of any adverse outcomes, however, it was associated with lower levels of baseline platelet count. Nevertheless, cluster 5 with completely positive for anti-Ro52 and a high rate of anti-gp210 was characterized by a high proportion of male patients and a low survival rate. It can be argued that cluster 5 represents a subgroup of patients of the pathological process underlying cluster 1, in which poor outcome had been proven for both clusters with a dominant anti-gp210 coincidentally.

In the present study, anti-SSA and anti-SSB was associated with PBC combined with SjS, while only anti-SSA correlated with low levels of baseline AST or ALT as well as high levels of IgG. High serum IgG levels are considered to be one of the serological manifestations of primary Sjögren’s syndrome (pSS) (28). Cluster 2 with the predominant antibody of anti-SSA and anti-SSB was characterized by a high proportion of SjS, a high rate of IgG >ULN, and a low mortality rate during the follow-up period, which could explain the ability of anti-SSA to play a major role in this cluster. PBC is known to have both hepatic and extrahepatic manifestations, and several other autoimmune disorders can coexist in patients with PBC (29). Herein, SjS was the most common extrahepatic autoimmune condition in 7.2% (n=36) of patients with PBC, while 16 patients were distributed in cluster 2. Surprisingly, previous studies have shown that when extrahepatic autoimmune diseases co-occur with PBC, the cases tend to be less severe; PBC is usually milder and occurs at an early stage (stage I-II at liver histology) in the presence of SjS (30, 31). The findings would explain the low mortality rate noted in cluster 2 until the date of the last follow-up.

It should be noted that the majority of the observational studies and randomized controlled trials (RCTs) have excluded PBC patients with co-existent diseases affecting the liver (18, 32). Although several studies have reported data on the real-world clinical management of patients with PBC (9, 33), similar data on the immune-serological research of PBC are few to date (7). The present study provides important novel evidence regarding the autoantibody profiles of patients with PBC, and PBC with coexistent diagnosis of disease affecting the liver, such as PBC-AIH overlap, as well as patients with PBC combined with several other extrahepatic autoimmune disorders. PBC-AIH overlap was found in 38 patients (7.1%) of the study cohort, and 4 of them were found in cluster 4, however, PBC-AIH overlap was not the over-represented clinical feature of any clusters.

Currently, NAFLD is the most common liver disease, which is mainly associated with the incidence of metabolic syndrome. During the development of inflammation, NAFLD may evolve to non-alcoholic steatohepatitis (NASH) and eventually to cirrhosis and its complications; however, the involvement of the biliary ducts is rare (34). Hindi et al. (35) indicated that NASH and body mass index (BMI) ≥25 were associated with severe biliary duct damage and fibrosis in patients with PBC. NAFLD was mainly found in the patients of cluster 3, who exhibited better disease prognosis. Although UDCA may be effective on both of the diseases (36), the results are somewhat counter-intuitive. Because the co-existence of two chronic liver diseases, particularly those that predominantly affect different regions of the liver lobule, would be expected to result in more severe and progressive liver disease than when only one disorder is present (37). The effects of NAFLD on the prognosis of PBC require further assessment.

The present study has several limitations. Firstly, it is retrospective and subject to limitations in the relevant study design. Secondly, the medical records of inpatients with PBC were reviewed at a single hospital in northern China. Due to incomplete clinical data of outpatient with PBC, asymptomatic patients who would subsequently develop PBC-related symptoms were not investigated in the present study. Thirdly, patients with co-existent diseases affecting the liver and several other autoimmune disorders were included which may result in bias of the subjects. Finally, the final classification scheme needs to be combined with clinical experience and practice. Based on the known link with clinical factors reported in the literature and the clinical experience regarding the cognitive heterogeneity of the disease, such differentiation could be informative but need to be verified in the future for the clinical practice in terms of both prognosis and treatment planning.

## Conclusions

By using a data-driven statistical approach in a relatively large PBC cohort from a single center in China, five subsets of patients with PBC were characterized by different autoantibody profiles, clinical features, and prognosis. More critically, we confirmed the prognostic value of anti-gp210 for both liver-related death or liver transplantation, as well as adverse outcome especially in patients with cirrhosis by multivariate analysis under real-world conditions. It is evident that autoantibodies are widely available and autoantibody profiles of autoimmune diseases can still be useful for classification and predicting outcomes in the clinic.

## Data availability statement

The original contributions presented in the study are included in the article/supplementary material. Further inquiries can be directed to the corresponding authors.

## Ethics statement

The studies involving human participants were reviewed and approved by Beijing You’an Hospital, Capital Medical University. Written informed consent for participation was not required for this study in accordance with the national legislation and the institutional requirements.

## Author contributions

Conception and design: D-TZ. Administrative, technical, or material support: H-PY, YZ, H-YL, Y-ML, YH, H-P Z, C-Y H, X-H L, and J-L L. Patient’s follow up and clinical data collection: D-T Z, C-YH, W-MZ, YZ. Data analysis and manuscript writing: D-TZ. Get research funding: D-TZ, YZ, H-PY. Supervision and manuscript revision: H-PY and YZ. All authors contributed to the article and approved the submitted version.
